# INBED: A Highly Specialized System for Bed-Exit-Detection and Fall Prevention on a Geriatric Ward

**DOI:** 10.3390/s19051017

**Published:** 2019-02-27

**Authors:** Nico Jähne-Raden, Ulf Kulau, Michael Marschollek, Klaus-Hendrik Wolf

**Affiliations:** 1 Peter L. Reichertz Institute for Medical Informatics University of Braunschweig—Institute of Technology and Hannover Medical School, D-30625 Hanover, Germany; michael.marschollek@plri.de; 2 Institute of Computer Engineering, Technical University of Braunschweig, D-38106 Braunschweig, Germany; kulau@c3e.cs.tu-bs.de (U.K.); Klaus-Hendrik.Wolf@plri.de (K.-H.W.)

**Keywords:** health-enabling technology, geriatric, fall prevention, wireless sensor network, medical wearables

## Abstract

Objective: In geriatric institutions, the risk of falling of patients is very high and frequently leads to fractures of the femoral neck, which can result in serious consequences and medical costs. With regard to the current numbers of elderly people, the need for smart solutions for the prevention of falls in clinical environments as well as in everyday life has been evolving. Methods: Hence, in this paper, we present the Inexpensive Node for bed-exit Detection (INBED), a comprehensive, favourable signaling system for bed-exit detection and fall prevention, to support the clinical efforts in terms of fall reduction. The tough requirements for such a system in clinical environments were gathered in close cooperation with geriatricians. Results: The conceptional efforts led to a multi-component system with a core wearable device, attached to the patients, to detect several types of movements such as rising, restlessness and—in the worst case—falling. Occurring events are forwarded to the nursing staff immediately by using a modular, self-organizing and dependable wireless infrastructure. Both, the hardware and software of the entire INBED system as well as the particular design process are discussed in detail. Moreover, a trail test of the system is presented. Conclusions: The INBED system can help to relieve the nursing staff significantly while the personal freedom of movement and the privacy of patients is increased compared to similar systems.

## 1. Introduction

In this article, we show a highly specialized solution for a modular, inexpensive and medical needs-fitting system for fall prevention and detection, called INBED. The system can also detect restlessness and entering of risk areas while focusing on geriatric patients. Although intended to prevent, the INBED also detects fall events in the worst case. To the authors’ knowledge, this work presents the first system which combines this comprehensive set of functions as a closed system for clinical solutions.

### 1.1. Motivation

In German hospitals, the average fall ratio of patients is 4.7 falls per 1000 bed days [1]. With regard to geriatric wards, this increases to 9.1 falls per 1000 bed days [1], which has been validated by the quality report benchmark in geriatric health care from 2007. In the report, a fall ratio of 9.6 falls per 1000 hospital days was observed [2]. Many falls occur as a result of rising events in the immediate vicinity of the bed [3]. Although high-fall-risk patients are encouraged to call the nurses for help if they wish to get up, this directive is not always followed (e.g., due to cognitive impairments). The situations in outpatient areas are similar and again most of the falls occur during rising events for transitions.

Causes of increased risk of falling of elderly people are mainly chronic diseases (e.g., cardiac arrhythmia, dementia, Parkinson’s disease), predisposing factors like gait and balance disorders, visual impairments and vigilance disorders [4]. Patients with the diagnoses of dementia and motor deficits fall twice as often as patients with normal cognitive abilities [5]. A differentiated assessment of one’s own mobility often does not seem easy when cognitive performance is impaired [6]. The diagnoses of disorientation and dehydration are particularly important in the case of nocturnal fall events [7]. Furthermore, elderly people often suffer from increased frailty and multi-morbidity. The general likelihood of falling for over 65s is at least 30%,which was found in a recent study (men up to 43%, women up to 30%) [8]. Thus, the vulnerability and risk of falling injuries are particularly high. In sum, an increased tendency to fall is associated with a poly morbidity, as is predominantly found in geriatric patients.

Especially for elderly persons, one of the most common consequences of a fall is the femoral neck fracture [9]. While the probability of the femoral neck fracture increases moderately between the ages of 60 and 70, between 0.06 and 0.08%, it increases rapidly after the age of 70 to over 3%. According to current forecasts, the proportion of injury-related hospital stays will probably increase by more than 50% by 2050 due to demographic developments [10,11]. Furthermore, 10–20% of these patients will suffer mobility impairing fractures [12].

Beside their mental and physical burden, fall events are associated with immense consequential costs. According to Stöckle et al., the direct follow-up costs of a fall related femoral neck fracture amount to an average of €25,000 [12]. In 2000, in the US, the cost of treatment for overthrow non-fatal injuries of over 65s was $ 19 billion. The costs for home care were not taken into account in the case of persistent long-term care [13].

It is estimated that overall annual treatment costs of up to €2.77 billion [14,15] are incurred on the basis of fall-related hip fractures in hospitals and rehabilitation centres, in Germany.

The fall-related costs are according to WHO between 8000 and $ 26,500, considering only the direct and hospital costs. The average economic cost can still be $ 40,000 annually (UK). According to WHO, these costs will continue to rise until 2040 [16].

With regard to the current trend in demographic change and the resulting increase in the number of elderly (chronically) ill and care-dependent people, it is necessary to develop strategies in everyday life, as well as clinically suitable and intelligent solutions for the prevention of falls. A promising approach is the use of sensor-based alert-triggering systems, which inform the nursing staff when a patient tries to get out of bed.

In order to establish a system that can effectively prevent falls in clinical environments (in Germany), this must meet different requirements. Of course, the system must be sensitive and specific to the fall prevention function, but this is not enough for clinical use. The potential system must be intuitive in the clinical structure, as well as fit in with the work processes of the staff in order to be accepted. This also includes appropriate energy efficiency as well as the suitability of clinical approval, with respect to e.g., hygiene or EMI compatibility. Within this work, we show how to prepare a general fall prevention system for a clinical ward usage. The focus for this is on the usability and the intuitive use of the overall system within the daily care at the ward. Furthermore, we show how the rather easy but highly robust wearable core functionalities (based on a threshold algorithm using an accelerometer) works.

### 1.2. Related Work

Bed-exit detection systems for reducing patient falls are fundamentally available in two types, the ambient and the body-worn variants. These, in turn, can occur in various forms [17], which may be mattress pad systems or ground pressure mats, infra-red systems or cameras and garment clipping sensors (see [18,19,20]). The supporting function of bed-exit systems has been studied for several years. The positive influence on the fall rate of elderly persons affected by the accident has been demonstrated in various studies [21,22].

Depending on the diagnoses and symptoms of the patients, different advantages and disadvantages of the respective systems have to be identified. Among other things, extreme weight of an impacted person can adversely affect the adequate function of sensors. For example, if a patients weights under 50 kg proper function of pressure sensors is not guaranteed so that mattresses or ground pressure mats (cf. [21]) are useless. In addition, mobile dining tables or suitcases can lead to false positive alarms by standing on such floor sensors, which can affect a discontent of the nursing staff. Moreover, special cleaning procedures of nursing and/or clinical facilities can lead to the exclusion of such system components.

As already mentioned in Section 1.1, one of the main causes of increased risk of falling is dementia, which is often associated with underweight and restlessness [23]. For this reason, the implementation of a bed-exit system should be suitable to the diagnoses and symptoms of the particular patient condition. This is also in line with the findings of the National Institute for Clinical Excellence; “To be effective, they (Bed-Exit Alarms) need to be implemented with care and with a clear understanding of their limitations” [17].

For the general surveillance of patients independent from location (public or private), video monitoring is a proper solution (see [24,25]) but besides legal restriction, related to the usage of camera recordings in public, the basic acceptance is an important field. The acceptance of cameras for surveillance impartial from reason is not quite high in a major group. The acceptance differs under varying circumstances, but is in general under 50% (see [26,27]).

The Peter L. Reichertz Institute for Medical Informatics (PLRI) has been concerned with the possibilities of bed-exit systems for years. This culminated in an extensive clinical study with the aim of measurement of the overall reduction of falls on a geriatric ward, by close monitoring with a portable proprietary sensor system (shimmer sensor system [28]). For this purpose, a bed-exit alarm with this wearable was developed as a core component, which reliably detects rising attempts [22].

Within the study, a comprehensive requirement analysis in close collaboration with nurses and physicians revealed important requirements and preferences. Through an incremental design process, which includes laboratory tests and re-testing with some geriatric patients, a reliable prototype of a total system could be generated. The result of this process was a scalable technical solution, which proved its reliability in a one-year pilot study at a geriatric ward. As a result, it was found that the unobtrusive system worn on the subject body is convenient to use. The developed bed-exit system of the PLRI was tested at this time with 98 patients [22] and forms the background for the work at hand.

The usage of accelerometers is quite common in the field of fall detection or fall prevention [29,30,31], but unfortunately it is still in the stage of basic research of feasibility or the determination of accuracy and not in the stage of implementation of an overall system. Within this work, we show how a possible general and modular fall prevention system could work for the strictly regulated German clinical field.

## 2. System Overview and Scenario

**Figure 1:**
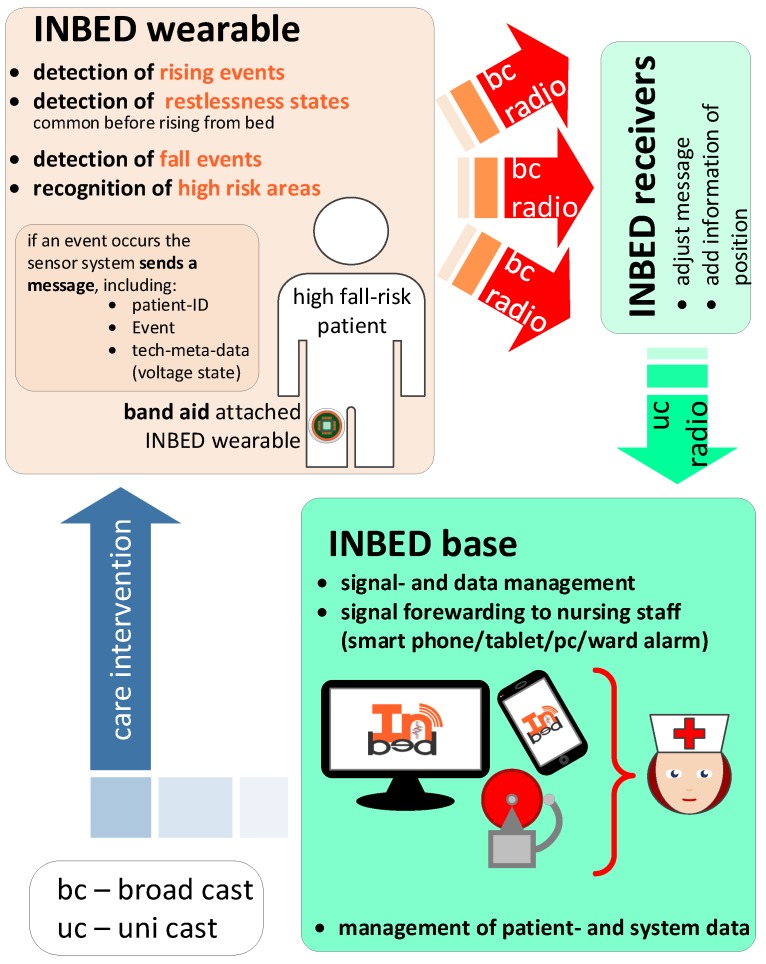
INBED system functionality diagram.

The entire INBED system is a highly specialized, modular prototype, developed by the PLRI and the Institute for Operating Systems and Computer Networks (IBR) with permanent support by clinical partners, above all the Municipal Hospital Braunschweig, Germany. The core unit of the system is a small, affordable wireless sensor board, the INBED wearable. Later on, this wearable is described in detail in Section 2.3. Here, the general functionality of the system is illustrated separate from system component details in Figure 1.

The patient wearable detects rising attempts and sends a signal to the nursing staff immediately. Thereupon, the nursing staff can help the patient to get up and assist the walk (e.g., for the toilet). By informing the nursing staff in appropriate time, and the accompanying provision of assistance during the on-the-spot treatment, it is possible to reduce falls in this area. Thus, the INBED system can eliminate the common belt systems or bed rails used on patients with high risk of falling while keeping the patient’s personal freedom of movement without compromising the patient’s sense of safety.

The wearable component of the system, the INBED wearable, will be attached to the patient’s upper leg on the upper half of the thigh (details will be described in Section 2.3.3). This position is optimal to detect rising event by angle changes of the legs without excessively affecting the patient’s comfort. Besides the wearable, the developed system contains several components like relay stations and a central signal processing base station to create a scalable communication network.

The interrupts of the wearable system immediately generate messages which include information about the current actions of the device suspender as well as the wearable component itself. In each generated message, the contained information, like device-IDs, are pseudonymized and encrypted by bit-shifting processes.

**Figure 2:**
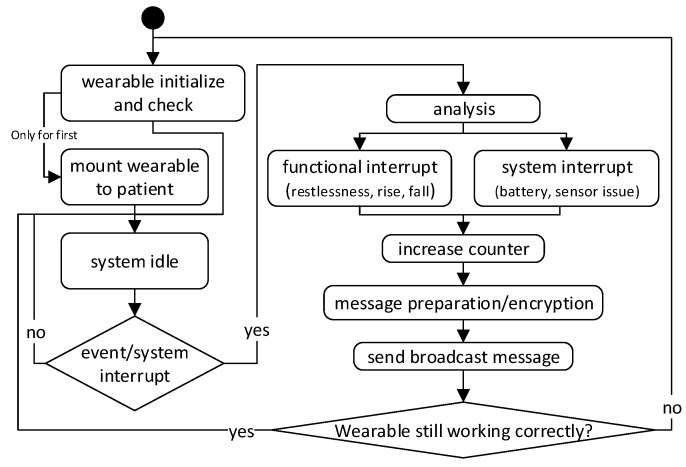
Flowchart of the INBED wearable internal process.

A wearable broadcasts generated messages to the network which are forwarded to the central base station. Figure 2 shows a basic flowchart of the general wearable internal process. Not all special cases of the function are shown in this model. The actual determination of movement events is analyzed directly on the wearable (see also Section 2.1 for detailed information of the functionality).

An intermittent node will update (extend) the message status and adds information on receiver assignment and receiver distance to allow a multi-hop communication via downward routes.

By the relay nodes, the encrypted and pseudonymized message is now directed, forwarded by using unicast (UC) to the system’s base station, which evaluates the message(s) and generates a corresponding alarm. The alarm is displayed (optically, acoustically and/or haptically) on the user interfaces device (HW-enhanced mobile phone) and the terminals (base station).

**Figure 3:**
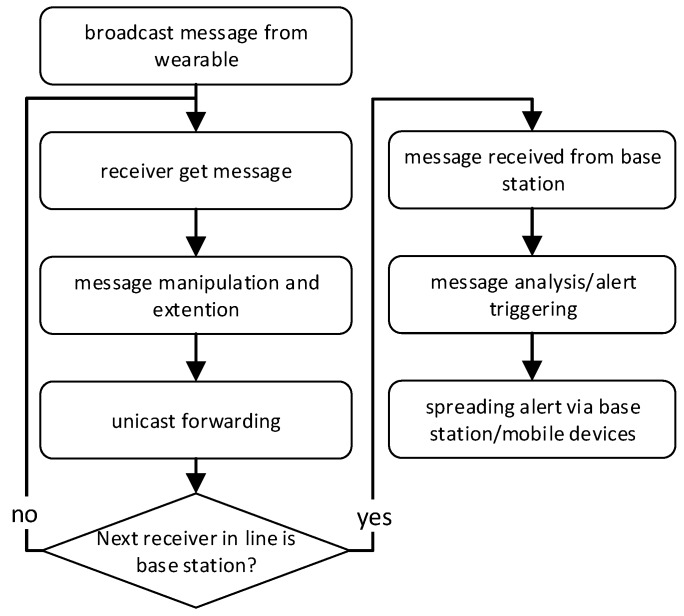
Flowchart of the general INBED system process.

In Figure 3 the flowchart summarizes this general system main process.

### 2.1. Clinical Scenario

For a better description of the system and its functions, we will use a fictional but typical clinical scenario.

The setting: A patient in a geriatric ward (high fall risk, motoric functional deficits but self-sufficient) is instructed to contact the nurses using the nurse-call-button in case of pain or other needs. Due to precariously walking, the patient should not go to the toilet alone. According to experience, geriatric patients do not always want to wait for the help of nursing staff for a variety of reasons. They are therefore unnecessarily in danger.

Hence, in this scenario, the patient is equipped with the INBED wearable, which has been attached to the thigh by means of a regular band aid, at admission to the ward. A radio receiver (relay node) has been installed in the room where the patient is located, as well as at certain distance on the ward floor (approximately every 20 m), and at strategic points in the ward, like exits, elevators or public common area and the drug delivery, to form a communication network. The INBED base station is located in the nursing room and connected to a ward staff computer. In addition, mobile devices are available as an INBED interface for the nursing staff.

**Figure 4:**
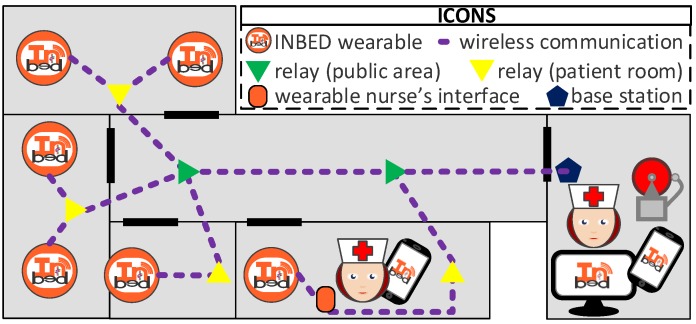
Floor plan of an exemplary ward with installed INBED system communication network.

Figure 4 shows a model of an exemplary ward equipped with the INBED system.

In the model, several patient rooms, as well as a nurse’s room and the ward hallway can be seen. In every patient’s room lays at least one patient, wearing an INBED device. The nurse’s room is equipped with several alarm interface devices, like a ward computer and the internal ward patient alarm system.

Furthermore, the general communication paths of the INBED system are shown in the model, including broadcast (BC) communication of the INBED wearable to fix patients room relay nodes as well as the UC communication between the relay nodes and the base station.

At the lower middle of the figure a special event is shown. A nurse is close to a patient, equipped with a smart phone and a system interface, which is utilized as a mobile relay node.

**Figure 5:**
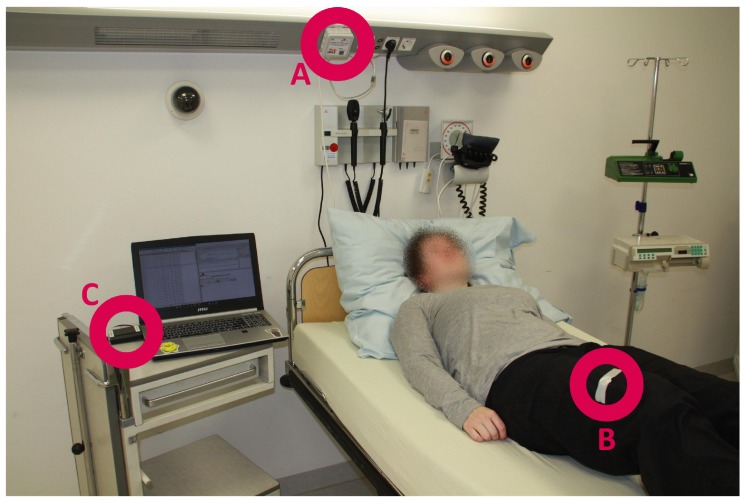
Structure of the INBED system (A: relay node, B: wearable, C: base station).

Figure 5 shows the setup of the INBED sensor system (for test purposes). The B labelled circle shows the INBED wearable, which is attached to the thigh of the test person by using commercial, medical adhesive tape. Circle A marks one relay node on the supply rail at the head of the station bed. The C circle marks the base station. Of course, for daily usage, the base station will be placed in the rooms of the nursing staff. The additional laptop was used for debug logging and convenient evaluation.

The procedure: For the scenario, the INBED patient will wake up in the night due to a strong urinary urge. A certain degree of physical restlessness is associated with the urge to urinate, so the patient begins to move in bed. This unusual high variance of the movement of the patient triggers a restlessness event. This event is now sent via a non-directional message and received by the patient-room relay node and contains the information described in the Section 2.3. The room-relay node manipulates the message in two ways. First, it marks the message as known in the communication network and, second, the relay node’s ID is added. At the same time, a confirmation of receipt is sent to the wearable, which again falls into an idle state. The modified message of the relay node is now directed towards the base station and may pass through various intermediate relay nodes. These intermediate recipients forward the message, always related with a confirmation.

When the message reaches the base station, the message parts are pushed into a database and used for generating a web application alert. This alarm is displayed prioritized with the database’s join information, affected patient (name/abbreviation), room and event type. The alarm information is displayed on the ward computer, as well as on staff’s mobile devices.

The nursing staff are now informed about the patient’s restlessness and decides to ignore this alarm for the time and continue with the current work and then look for the affected patient. The restlessness alarm can be deactivated by the staff at the ward computer application as well as on the mobile device.

The patient will trigger further restlessness alarms if the urge to urinate persists. This alarms can be notified by the nursing staff. In this way, the staff obtains knowledge about the urgency of the intervention regarding the patient.

Finally, the patient tries to get up and walk alone to the toilet without an active call for help. This triggers a rising-up-event which is distributed according to the restlessness event. At the base station, the rising-up alarm is triggered with a higher priority compared to the restlessness alarm, because the probability of a potential fall event is higher when a patient already exits the bed.

Due to multi-morbid and motor-functionally restrictions, the rising-up of a geriatric patient from a reclined position will usually take some time (cf. [32,33]).

Hence, rising is a medium-term process happening as follows:

1. rising the upper body
2. approaching the bed edge
3. twisting out the legs
4. moving the hip to the edge of the bed
5. placing the feet on the floor
6. the actual standing up.

With the information about the patient’s attempt to get up, the nursing staff are called to go to the patient as soon as possible. The alarm can not be canceled via application buttons.

When the nursing staff arrives at the patient, they can take care of his/her needs (accompanying walk to the toilets etc.). By measuring the received signal strength indicator (RSSI) of the wearable’s signal, the proximity to the deletedthe nursing staff, respectively the mobile device, can be estimated. An existing alarm will be switched off when the nurse arrives to see the patient and a potential new generated alarm will be prevented for a certain time (30 s).

### 2.2. Special Characteristics of the System

For the particular use case of the INBED system in clinical facilities, the specific characteristics of such environments have to be taken into account. This includes requirements of hygiene, power management (related to the patient retention time), legal restraint and properties that enable or support the logistics and administration (e.g., EMI compatibility). For this work the requirements analysis was realized by several surveys and observation visits of the clinical wards. With the obtained information two groups of requirements on a clinical fall prevention system could be determined, the *specific* and the *non-specific* requirements.

The specific requirements come from the needs of the clinical daily routine including system properties like the waterproofness of the wearable. Hereinafter all determined specific requirements are listed and explained.

The first system property which the nursing staff had mentioned, is the *type of alarm* of a concept fall preventing system. The alarm has to differ from other existing alarms like patient call alerts or alerts triggered by ward devices, like an ECG. Furthermore, a cascading alarm with different signalizing ways, like acoustical, optical or haptic alerts, and distinct alarms of occurred events (rising, restlessness and fall), is wanted.

Another important requirement is the *size of wearable components* within the INBED system. This includes the wearable which should be mounted on patients, as well as the wearable relay node carried by the nursing staff. For the patient wearable a general minimal size is given by the chosen battery (we use the commonly used, cheap CR2032 coin battery, diameter 20 mm). For the nursing staff’s interface the basic acceptable magnitude is only restricted to a least possible height of the device. We achieved, with a based case, a height of about 6 mm. For the staff wearable the personnel wished for a small, but not tiny device, because they are afraid of losing it. We decided to use common smart phones and add the functionality of IEEE 802.15.4 (version 2003/2006/2011) radio communication, as the low power RF transmission (BLE) is not allowed at participating clinical wards. We use the IEEE 802.15.4 to create a simple modular network especially for the system components, that is mainly independent from the clinical communication infrastructure—this is a requirement for potential upcoming implementation (e.g., as a medical device).

Moreover, the health care professionals described that wearable devices are affected by harsh conditions in daily clinical routine. Related to this, the requirement of *waterproofness* is caused by the usage of liquid detergents or potentially incontinent patients. This is also considered by the choice of the housing.

Another highly important property mentioned by the nursing staff is the *reliability* of the system. The determined statement is, that the system has to provide a sufficient zero-missed-threat-ratio, related to rising events. Besides, a minimal false positive rate of event alarms is expected. From further projects, a ratio of two false-positive alerts per night is determined as acceptable.

After the development of a first conceptional prototype, we discussed the basic technical properties with IT-specialists, including the CIO of the participating hospital, to obtain detailed information about technical restrictions, like *communication protocols*. This results in a modular expanding communication network using IEEE 802.15.4 radio and a optional interface for a potentially available WiFi.

Besides the specific needs, several non-specific requirements are determined and evaluated with help of clinical professionals. These non-specific properties are claimed, but not explained by the nursing staff in detail.

One frequently mentioned requirement for the INBED system is the *usability*. By this, not only the ease of use of the software is meant, but simple application in the clinical daily life. For example, the intuitive mounting of the patient’s wearable or the compatibility of the wearable case with the common sterilizers. Related to this, *wearing comfort* and *inconspicuousness* of all wearable devices are of importance.

Furthermore, the *sensitivity* is an important concern as already mentioned within the false-positive alert ratio requirement.

At least the actual *costs* for the overall system should be as low as possible. The *maintainability* is also among the costs. This includes remote administration, debug logging, as well as support of internal functions like self-randomization or self-pseudonymization.

### 2.3. INBED Wearable and General System

The INBED sensor is the core component of the entire system described in Section 2. To measure activities and movement with high accuracy but without any loss of comfort, it takes a well-engineered wearable device that can be attached to patients. Although there is a plethora of existing wearable sensor systems, the existing platforms are not well-suited to fulfill the tough requirements of clinical environments.

To guarantee the freedom of movement, the wearable has to transmit its data wirelessly while relying on a mobile energy source, in particular, a battery. The size of the wearable should be fairly small as the wearer should not be bothered. However, this implies only a small battery with limited capacity. As a consequence, the entire design of the hardware and software is driven by the demand of energy efficiency. As sanitary regulations will not allow to simply replace batteries, the sealed wearable has to operate for potentially long lifetimes, e.g., the period of treatment which is near 14 days by mean. The costs are another limitation for the design of the hardware. For the desired use-case, a disposable-wearable is intended.

In the following, we present the hardware architecture of this application-oriented wireless sensor node. Moreover, the features of the software implementation of the node are discussed.

#### 2.3.1. Hardware Architecture:

**Figure 6:**
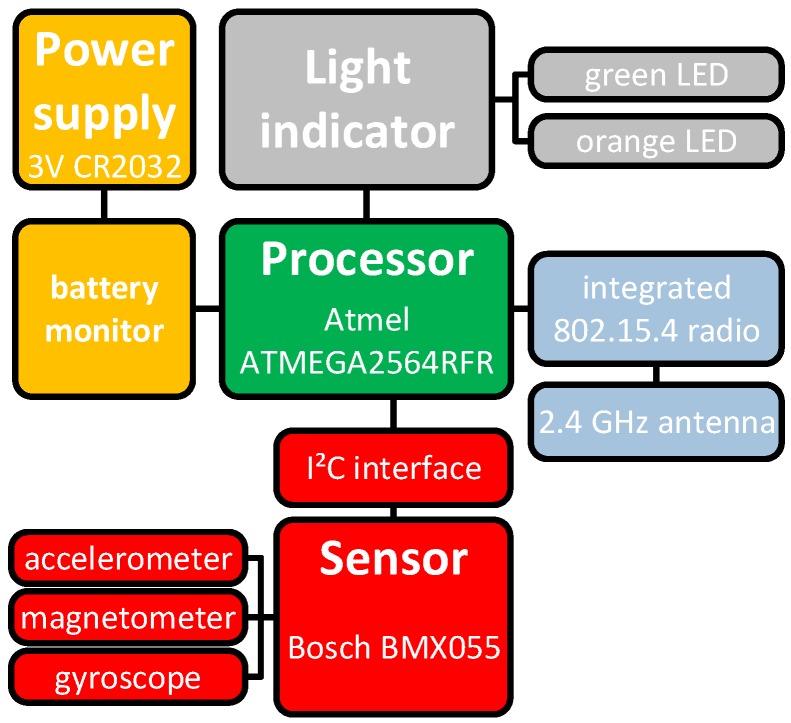
Block diagram of the hardware architecture of the INBED wearable.

Figure 6 shows a block diagram of the INBED’s wearable hardware architecture. The design of the INBED wearable is related to the classical architecture of wireless sensor nodes. A processing unit, represented by an micro controller unit (MCU) executes the software and controls the other parts of the nodes, in particular, the transceiver unit for wireless communication and the sensing unit for gathering the movement data.

Processing and transceiver unit: In this case, we used an ATmega2564rfr2 System on Chip (SoC) (Atmel Corporation, San Jose, CA, USA) where the MCU as well as the transceiver unit are fully integrated on a single chip. This offers several advantages in terms of size, performance and costs. In the first instance, the footprint of the INBED is reduced significantly. Moreover, the performance of the communication between the MCU and the transceiver is increased, as registers are used as a communication interface. Compared to classical node designs, where the transceiver is integrated by using bus-interfaces, the shared register communication is much faster.

The ATmega2564rfr2 includes both, a low-power 8-bit MCU based on the AVR enhanced RISC architecture as well as a fully IEEE 802.15.4-compliant radio transceiver for the 2.4 GHz ISM band. To reduce the costs, a simple PCB dipole antenna is used. With regard to the sensitive data that are collected and forwarded by the INBED wearable, this SoC also supports security features like AES hardware encryption and a true random generator. For high data rates, e.g., when raw sensor data should be transmitted directly, the transceiver also enables data rates up to 2 MBit/s.

Sensing unit: The selection of the sensor set of the INBED wearable is focused on the particular use case (cf. Section 2.1). We used a Bosch BMX055 (Bosch Sensortec GmbH, Reutlingen, Germany) [34] 9-axis sensor module. This module combines a three-axis accelerometer, a three-axis gyroscope and a three-axis magnetometer on a single SoC with a very small footprint. Thus, this module can be used to detect movement, rotations and magnetic heading in nine degrees-of-freedom. The BMX055 is connected to the ATmega2564rfr2 via Inter-Integrated Circuit (I^2^C)-bus. We use the BMX055 multi-sensor with a sample rate of 10 Hz. By using the accelerometer, the BMX055 implements the option of a free-fall detection which can trigger an interrupt. However, many other interrupts can be configured to trigger on several events, e.g., thresholds. Hence, by extensively using the pre-configurable interrupts, the energy efficiency of the entire INBED wearable can be increased as reactive programming models can be applied.

The INBED wearable is also able to measure the environmental temperature. Both, the ATmega2564rfr2 and the BMX055 have an internal temperature sensor.

Power unit and monitoring: The entire INBED wearable is directly powered by a standard CR2032 battery. Thus, the cost overhead and efficiency limitations when using voltage regulators are excluded. All components are designed to be supplied by a voltage level between 3.6 V and 2.4 V. However, the INBED wearable must be reliable and a missed detection of a fall-event is highly critical. To guarantee that the voltage level never undershoots the required level for reliable operation, the ATmega2564rfr2 integrates mechanisms like brown-out detection and a battery monitor. The voltage can be sensed continuously to allow an early notification when a battery runs out of energy.

**Figure 7:**
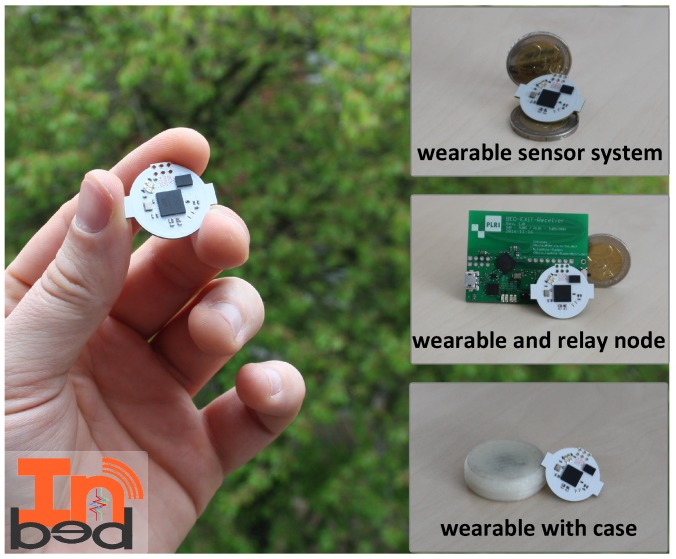
Current INBED wearable board with case and relay node.

All components are mounted on a two-layer PCB with the dimension of 20 mm diameter plus two 1 mm wings (22 mm maximal diameter) and 6 mm in height. In Figure 7, the INBED board, with and without case can be seen, including an image of a coin scale and in comparison, with the relay node.

The total costs for one are approximately $ 15 (QTY 1 K). In sum, the INBED wearable is cost, size and energy efficient and therefore ideal for bed-exit detection and fall prevention in clinical environments. The INBED hardware is open source and we provide schematics and EAGLE-files [35].

#### 2.3.2. Software Implementation

**Figure 8:**
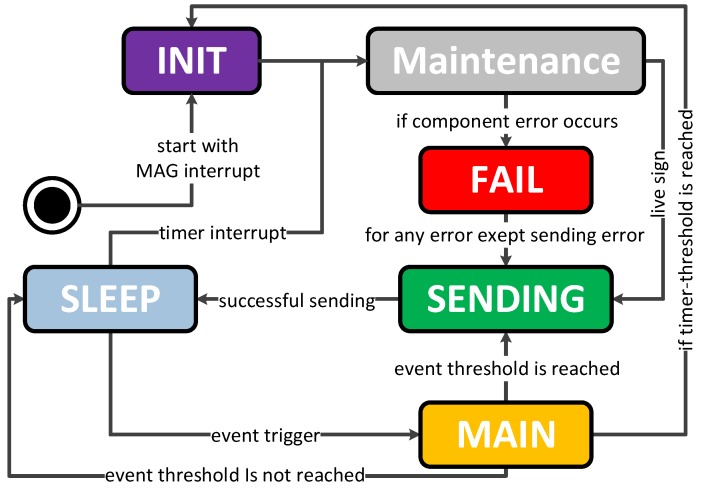
Current INBED wearable state chart diagram.

Besides the hardware, software and its development is a basic part of the whole INBED system solution. Basically, the programming model for the software is event-driven, which allows a highly reactive system while keeping the energy demands of the INBED wearable low. Figure 8 shows a state chart of the software implementation.

As can be seen, the INBED wearable internal structure starts always with *INIT*-state. Within this state, it is checked whether the wearable is initially started or already been used.

If it is first booted, the magnetometer interrupt of the BMX055 multi-sensor is activated and all components of the INBED wearable are set to sleep state for power saving proposes. By this set-up, the wearable can be stored with minimal loss of battery voltage. To leave this state, the device can be activated with a common magnet. In run mode, the *INIT* will always forward the process to *MAINTENANCE*-state, in which the board components (e.g., BMX055) will be self-checked. If a failure occurs, *FAILURE*-state will be reached and a signaling LED is activated. In both cases, the *SENDING*-state will be reached and a corresponding message will be created and sent if possible. For more details, see Section 2.3.4.

After a successful sending event, the wearable will go into the power saving *SLEEP*-state again.

The *SLEEP*-state can only be left by an occurring interruption. However, these interruptions can be triggered by several events, e.g., for rising from bed, for restlessness (for details see the following paragraph for the different possible interrupts) or a regular timer interruption. The event interruptions lead to the *MAIN*-state in which the specific events are processed. If a predefined threshold for an occurred event is reached, the *MAIN*-state will trigger the *SENDING*-state. With regard to movement events, the events are prioritized from restlessness (lowest and optional), over rise (obligatory), to fall (highest obligatory). The priorities can be adjusted to the needs and wishes of the nursing staff. The timer trigger is, in general, an advanced use of internal interruption structure of the MCU. With this function, the wearable internal Software (SW) will periodically be reset to the *INIT*-state, which results in a continuous self-check in *MAINTENANCE*-state. In the following the events and triggers are described in detail.

Restlessness Events: A patient’s restlessness will be monitored by an axis absolute value factor based on movement activity (derived from the accelerometer). If a weighted counter’s threshold is reached, an alarm will be triggered, otherwise, the counter will fade out to zero over time.

Again, the software implementation uses the internal interrupts of the BMX055 to aggregate the move time. The used time period is eight seconds each. While the used threshold for weighted interrupt’s counts is at 40. The fade is realized by the follow setting:

$$\begin{array}{l} count_{t}=\sum_{i=1}^{40}restless\;Interrupt_{i}\;\cdot \;w;i=interrupt\;occurring\;within\;time\;period,\;w=weight\\ \Delta_{count}=count_{t-1}-count_{t};t=time\;period\\ \left\{f_{Restless}=\Delta_{count}*\frac{2}{3}|\Delta_{count}>0\right\} \end{array}$$

Rise Events: This method utilizes the internal interrupts of the BMX055. A BMX055 internal interrupt is triggered when the position changes from “portrait” to “landscape”. To utilize this interrupt function, we adjusted the triggering angle threshold according to the first utilized method, using linear angle calculation done by arctangent, over all three axes.

$$A_{x}=arctan\left(\frac{x}{\sqrt{y^{2}+z^{2}}}\right);\;example\;for\;x(other\;axes\;are\;similar).$$

Within the *MAIN*-state, a weighted counter will be increased by each rising interrupt. The event trigger will be fired whenever the counter overshoots a predefined angle threshold of the accelerometer. Thus, sending of events for only temporal angle variation, e.g., related to a patient’s bed position changing, is avoided. The used angle to trigger the interrupt is at $63^{\circ }$–$117^{\circ }$ for rising. The counter will be fading out over time. The fading procedure is equivalent to the described restlessness count fading above.

Fall Events: By reaching a near zero-g threshold in acceleration, a fall event is very likely.

$$\begin{array}{l} accelearation\;Vector\;\left[g\right]=\sqrt{{\left(\frac{v_{x}-o_{x}}{s_{x}}\right)}^{2}+{\left(\frac{v_{y}-o_{y}}{s_{y}}\right)}^{2}+{\left(\frac{v_{z}-o_{z}}{s_{z}}\right)}^{2}};\;is\;neutral\;position\;1\\ v=value,\;o=offset,\;s=scale \end{array}$$

This equation to calculate the overall acceleration is done by using the 3D-ellipsoid equation, based on the Pythagorean theorem (see [36]).

In addition, when a zero-g phase is followed by a sudden high shock, the fall event is triggered. However, if the duration of a free-fall is only fairly short, no event is triggered to avoid false-positives.

Again, the implementation utilizes the BMX055’s internal interrupts to optimize the power consumption. In particular, the free-fall interrupt is utilized for this case.

hlRisk Area Detection: The detection of a patient entering a previously defined risk area is formally not done by the INBED wearable, but by the stationary relay nodes. For this a relay node has to be declared as a “risk area relay node”. If a message is first received by the “risk area relay node”, an alert will be triggered. The accuracy can further be improved when the relay nodes measure the RSSI to estimate the distance of the INBED wearable to a specific relay node.

Failure Events: Besides the patient-related event messages and the periodic “life sign” message, failure events may occur and will result in a message to the base station system.

The battery voltage is attached to every message that is sent to the base station anyway, but when the battery monitor interrupt of the MCU is triggered, an additional failure message will be dropped. A sensor failure is detected by *MAINTENANCE*-state’s check up. Of course, radio failures can not be triggered by the INBED wearable directly. This type of failure is triggered at the central base station due to the absence of “life sign” messages.

For debugging purposes, all events are stored in administrative log at the central base station together with patient-related data.

#### 2.3.3. Wearable on the Patient

**Figure 9:**
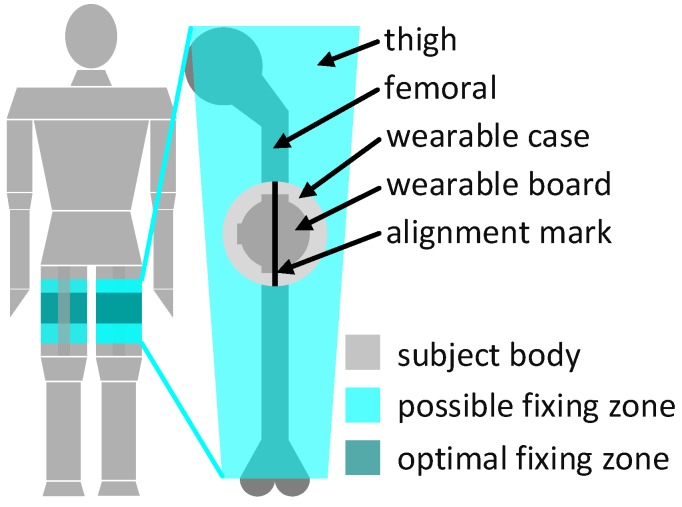
Diagram of the mounting position on a patient with detailed mounting position on patient’s thigh.

The wearable component of the system, the INBED board, will be fixed at the patient’s upper leg on the upper half of the thigh, as shown in Figure 9. The wearable fixing can be done by a regular band aid of a proper size.

The fixed position of the INBED wearable on the thigh of the patient was determined by numerous conversations and wearing tests.

Basically, it was clear that positioning near the body center makes sense in order to avoid strong accelerations, e.g., to avoid the extremities. Due to the often more bedridden patients, areas such as the back or the lateral hip have been eliminated as an option to minimize the risk of ulcer (pressure).

The positioning on the thigh makes it difficult to differentiate between sitting and lying, which is not a disadvantage for the challenge of future general fall prevention.

For the correct orientation of the accelerometer’s axis, an alignment mark is placed on the wearable case. The alignment mark must be oriented in the way of the femoral, as can be seen on the right side of Figure 9. The position around the thigh under consideration of the case mark is irrelevant, but for best comfort, a position on the outer front side in the optimal apply zone (see Figure 9, left) should be preferred. The interrupts of the wearable system ultimately generate messages which include information about the current actions of the device suspender as well as the wearable component itself.

#### 2.3.4. Communication Infrastructure

For each generated event alarm by the INBED wearable, various information will be sent. These are information about the origin (ID of the sender), the nature of the incident (code of the event) and logistic data for the processing or function provision (battery voltage, etc.). In each generated message, the following data are encrypted and pseudonymized by bit-shifting processes:

- message status (new or known in network)
- event (13 = restlessness, 14 = rising, 15 = fall, 16 = risk area)
- device-ID (unique key within a ward)
- battery voltage.

The overall system deploys a IEEE 802.15.4 communication network and is, therefore, not dependent on external infrastructure.

We decided to use IEEE 802.15.4 standard as a low power modular solution (see [37]). In this case Bluetooth could also be a proper solution, but was prohibited by the responsible IT office of the clinic.

The network is structured as a tree with the base station as root node, relay nodes as intermediate nodes and the INBED wearables as leaf nodes while the wearables for the nursing staff can join the tree at any point.

While the INBED wearables send their messages via BC, the relay nodes forward their messages via UC towards the base station.

Every message will be replied with an ACK. If the message is received by the base station, the contained data will be stored in a web application database which provides the information on several nursing staff devices (see Section 2.1).

**Figure 10:**
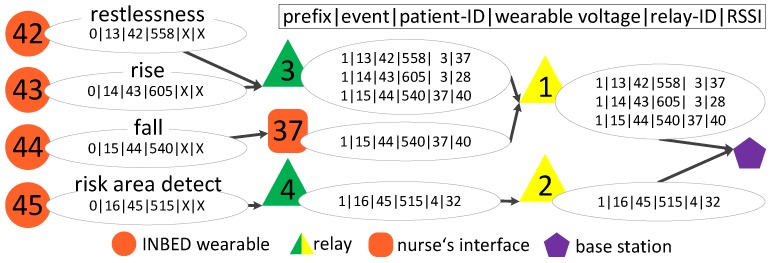
INBED system communication diagram.

Figure 10 provides the network and message structure of the INBED system. A message contains *message prefix*, which shows the state of the current message within the system structure (new or already known), event identifier, device ID (wearable device), battery voltage (wearable device), first relay node ID and RSSI (cf. Section 2.1). In the diagram, four different events are listed (restlessness started on device 42, rising—device 43, fall—device 44 and risk area detection—on device 45). The upper three events are connected to the relay node number three and the bottom device sends to relay node number four. Fall events are not solely forwarded to the base station but can be displayed by intermediate nursing staff nodes.

In sum, the communication infrastructure is adaptive and, thus, robust against link failures or relay node outages.

## 3. Results

The functionality of the INBED system was already tested by several trials from basic Hardware (HW) and SW tests to system-wide tests. These include the evaluations of the acceleration measurement, the communication and data management.

### 3.1. Communication Range

First, we investigated the communication range of the INBED wearables. This was evaluated by means of some devices. Therefore, INBED wearables were equipped with batteries and iteratively moved further away from a relay node. On average, a communication range of 25 m is reached for line of sight, while about 15–25 m are expected in a common building structure. The different results are related to the specific structure of the sending path’s obstacles, like walls, water pipes or power lines.

### 3.2. Energy Efficiency

To evaluate the energy efficiency of the nodes, we assume a battery power of 230 mAh (this is an average battery power, which can vary because of storage time), a clock frequency of 2 MHz of the MCU and a standard room temperature of less than 25 ${}^{\circ }$C. For the nurse’s interface, two “AAA”-batteries, with a capacity of about 1200 mAh, are assumed (this is an average battery power, which can vary because of storage time). In “worst-case”, which means a continuous transmission of a fixed INBED message every 0.9 s, the INBED wearable lasts about 52 h (approximate 200,000 transmissions) while the nurses’ device runs more than 164 h (approximate 630,000 transmissions). The sending procedure is by far the most consumptional function of the device, which meant that this is the battery limiting parameter.

The “best-case”, where only life-sign messages occur, results in more than 19 days lifetime for the INBED wearable and about 100 days for the nurses’ interface.

### 3.3. Functionality Test

The functionality test of the entire system was performed under realistic conditions. For this purpose, the system was set-up with all components and three test persons were equipped with an INBED wearable before different scenarios have been investigated. The test cases include both, intended triggering of events (e.g., by standing up, falling, restlessness, error triggering) and testing of the normal case without explicit supervision. For the tests the wearable was attached to the upper front of the thigh as described in Section 2.3.3.

To underline the suitability for daily use the test series were also performed in a clinical environment.

Restlessness Events: For the test of restlessness events, the subjects went to a lying position and then turn from side to side. The triggering of the corresponding event is expected during the repeated rotation process. Prior to the test runs, we performed a learning phase based on collected data of rising events from different subjects within a cross-validation.

For the restless detection six subjects were included (m = 2, w = 4, age = 28 ± 3). First we started with temporal trials which include the already described setup of several movements of the subject in a lying position. Within this the balance of detecting excessive movement by ignoring single position changing were performed. Furthermore six instances of two real nights of measurement while sleeping were performed with a camera reference.

For the first lab test only the high intense movements triggered a alarm. The nightly test should reveal the specificity. For those no false alarm was triggered.

**Figure 11:**
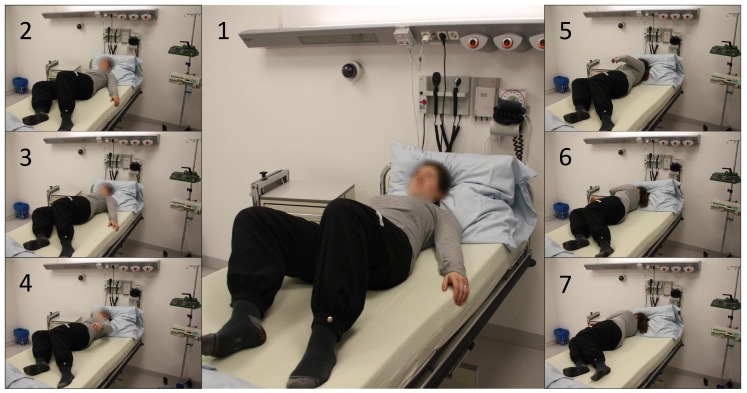
Procedure of a general restless phase, represented by lateral changes of position due to rotations of the test subject with equipped INBED wearable.

**Figure 12:**
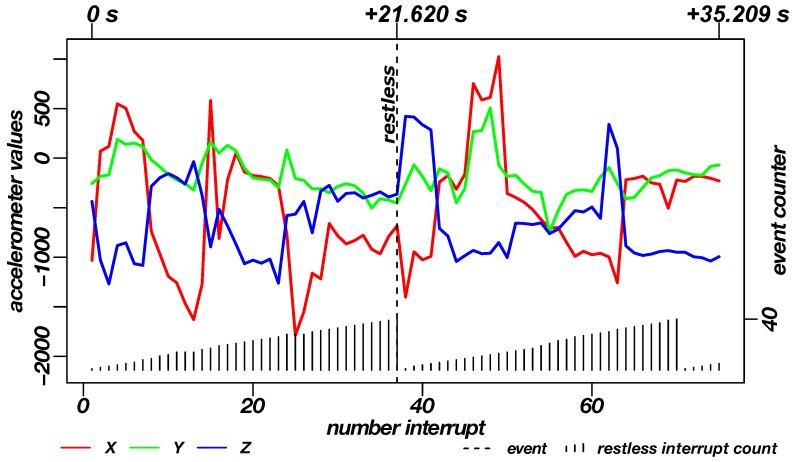
Diagram of the recorded data of the INBED wearables during a restlessness measurement on the subject. By means of the red-green-blue graphs, the recorded raw triaxial acceleration data of the sensor node can be seen for each interrupt of the wearable. Plotted in the lower area is the restlessness counter state, which counts up to the threshold of 40. The counts are weighted by the kind of movement. A dashed line shows the triggering of the restlessness alarm in the diagram. In the upper part the time interval of different milestones to the first interrupt is marked.

Figure 11 shows the data of the INBED wearables for one example of restlessness measurement. According to the person’s movement, interrupts are trigger the wearable to wakes up. Subsequently it reads out information about the three axes and the type of interrupts, e.g., if restlessness occurred. In the diagram of Figure 12, the raw acceleration data of X, Y and Z are shown in red, green and blue for every occurring interrupt, not as an linear measured sample. Furthermore, the restlessness event counter is shown in the lower part of the diagram. The counts of the this diagram are weighted by the kind of the movement related to the intensity of the motion itself (calm motion = 1, stronger/quicker motion = 2). The recording of the restlessness measurement is about 35 s long, with the actual alarm triggered after about 21 s especially for the circumstances of this restlessness event, which is not representative for every other hurry.

Due to the constant restlessness caused by the movement of the subject, the INBED system does not have the opportunity to let the restlessness count decay again by the fade out described further. Thus, the count is reset only after reaching the restless threshold.

**Figure 13:**
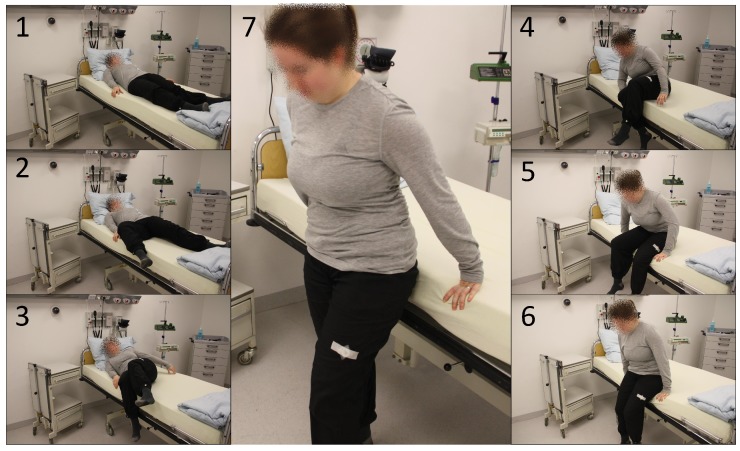
Procedure of a general rising out of bed, represented by seven phases from lying down over sitting on the edge of the bed to the uprising itself.

Rise Events: For the validation of rising detection, subjects started from a lying position and slowly began to rise. Finally, they stood up and got out of the clinical bed. In Figure 13 the general procedure of the uprising to a standing position is pictured. The first three phase images show the approach to the edge of the bed and the beginning of the uplifting of the upper body. The phase images 4 to 6 then show the overcoming of the bed edge with the body focus and the final raising to the standing position (no. 7).

The rise detection is the most important functionality of the general system, because of this we focused on testing the sensitivity. Since the first development for the clinical study (see [22]) the rise detection algorithm is only adjusted in terms of energy efficiency and to optimize the specificity (adjusting the angles to a more narrow interval—see Section 2.3.2—”Rise Events”). For testing we mounted the wearable on in total 224 subject while several measurements. The measurement were totally anonymous by means, no subject meta data was stored. For the setup every participant was told how the algorithm works and they should try to trick the system.

By those test we achieve a sensitivity of 100% in total only slight differences in detection speed were mentioned (few seconds).

**Figure 14:**
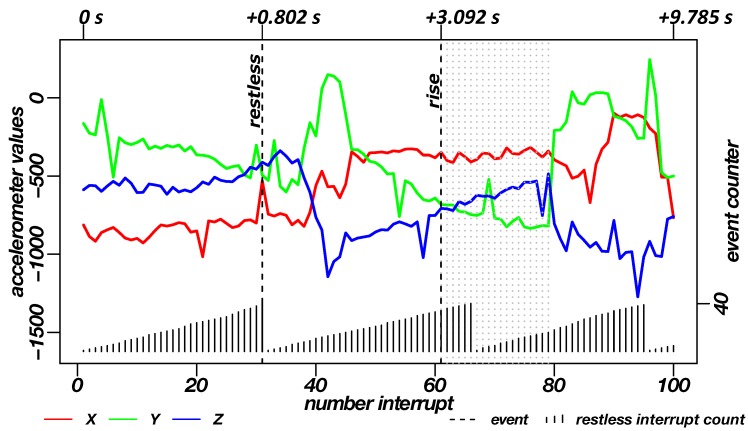
Diagram of the recorded data of the INBED wearables during a rising measurement on the subject. By means of the red-green-blue graphs, the recorded raw triaxial acceleration data of the sensor node can be seen for each interrupt of the wearable. Plotted in the lower area is the restlessness counter state, which counts up to the threshold of 40. A dashed line shows the triggering of the alarms (restlessness and uprising) in the diagram. For the rise event after the triggered alarm grey dotted lines are plotted to represent the standing. In the upper part the time interval of different milestones to the first interrupt is marked.

The diagram in Figure 14 shows an example of the data generated during the rising measurement. Here, as with the restlessness recording, the restlessness counts as well as the raw acceleration data of the three axes at the point when the interrupt occurred can be seen. The recording of the uprising process differs from the restlessness in terms of duration, this is due to the functional determinism of rising. With the standing of the subject, the rise up is complete. Thus, a measurement of a fixed period of time is not practical. In the shown case, the recording is about 10 s long. After about one second, the restlessness and after about three seconds the rising was transmitted as an alarm. The dotted lines in the diagram, right after the rise alarm, represent the occurrence of further rising/standing interrupts. From these interrupts, the standing of the subject can be derived.

**Figure 15:**
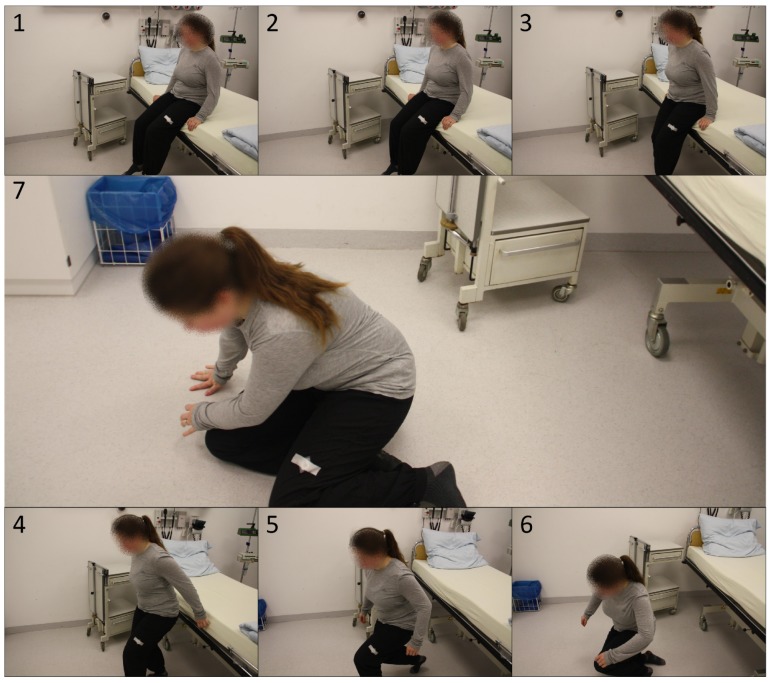
Procedure of a general fall, represented by a fall directly after an uprising.

Fall Events: For the validation of the detection of fall events, the corresponding alarm events were simulated within a clinical environment. Figure 15 shows the sequence of a fall measurement in seven phases. Images 1–3 show the last uprising moments to the general standing pose of the subject. In image 4, the subject moves a few steps away from the bed. From images five up to seven, the actual fall to the front is shown.

For the fall detection tests, six subjects were included (m = 2, w = 4, age = 28 ± 3). We performed several heavy falls from a standing position to rather hard and thin mattresses, for subject protection. The subjects, carrying two sensors at each leg, were asked to fall several times in different directions (front, right, left, back, side-front, side-back) and furthermore, to protect or intercept there fall at least as possible. We excluded “soft” falls from the test, because the danger for potential patients is limited and the trying of threshold based detection would lead to a lot of false-positives e.g., at walking. All of the heavy falls could be mentioned by the system, which means a sensitivity of 100%.

It should be mentioned that this sequence is an exemplary but representative fall event in clinical environments.

**Figure 16:**
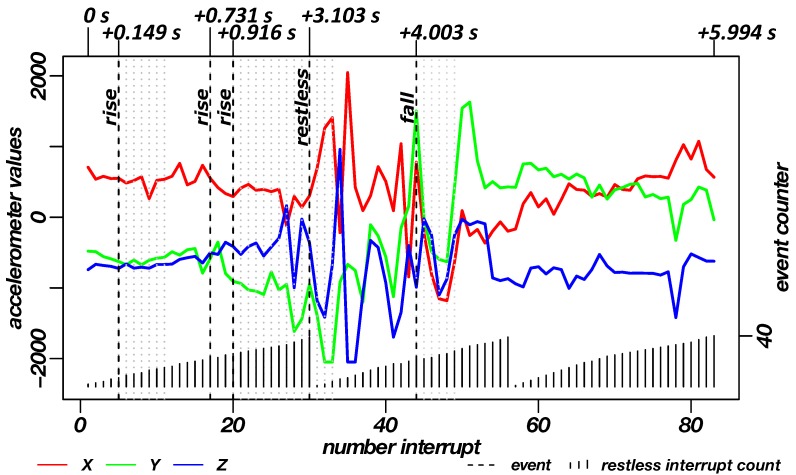
Diagram of the recorded data of the INBED wearables during a rising measurement on the subject. By means of the red-green-blue graphs, the recorded raw triaxial acceleration data of the sensor node can be seen for each interrupt of the wearable. Plotted in the lower area is the restlessness counter state, which counts up to the threshold of 40. A dashed line shows the triggering of the alarms (restlessness and uprising) in the diagram. For the rise event after the triggered alarm grey dotted lines are plotted to represent the standing. For the fall event after the triggered fall alarm light grey dotted lines are plotted to represent the further high/low-g interrupts. In the upper part the time interval of different milestones to the first interrupt is marked.

The associated data record for the fall is shown in Figure 16. Again, the raw acceleration data are coloured in red, green and blue (X, Y and Z axis) as well as incoming interrupts. The lower part of the graph shown the accumulation of restlessness events. The dashed lines in the plot show the triggered alarms of the three functions (restlessness detection, detection of rising/standing, fall detection). The rise detection can be followed by grey, dotted lines, which represent more rise/standing events that indicate a longer standing or as shown in the sequence several steps (cf. Section 2.3.2—”Rise Events”). The high impact of the fall event is followed by further high/low-g interrupts, indicated by light grey, dotted lines.

By imitating rises, restlessness and falls, optimal thresholds for the detection of events have been evaluated. For the test of the rising events, the best thresholds for the x-axis were determined to be $63^{\circ }$–$117^{\circ }$. For the fall events, an optimum value for the free-fall phase is ≤0.4 g and for the impact variance of ≥1.3 g.

Besides the described tests above, further trials have been performed. Four INBED wearables were attached to two subjects over two nights. An additional INBED wearable was not worn on the body for comparison purposes. The actual standing up was documented by a hand-written protocol. The unrest was documented by a camera recording of one person for both nights. The goal was to trigger as little false-positive event detection as possible. As a result, no false-positive events were recorded within this evaluation. Any larger movement (e.g., rotation) seen in the footage of the camera has also been recognized by the INBED system as a restlessness event. Any documented rising was detected by the system, as well. Again, no false-positive standing up was recorded here. However, problems were encountered during the imitated fall tests. Heavy stomping gait patterns and individual steps can also be detected as a false-positive fall by the selected thresholds.

Parallel to the described testing, several INBED overall system interviews and demonstrations, for health care professionals and clinical IT experts, were done to improve the system and the system settings.

The controlled error triggering is due to software manipulation on the INBED wearable. Error events include errors of the accelerometer, the failure of a message transmission and the occurrence of a low battery voltage. As expected, all system error messages were detected and displayed by the system.

## 4. Conclusions

Patient falls in clinical and nursing facilities are a serious problem, especially for elderly people. The aftermath of a fall affects relatives, clinical institutions, health care providers, health insurance companies and, finally, many members of society in a negative manner. Our close cooperation with the clinical nursing staff of the geriatrics ward, the municipal hospital Braunschweig, Germany, offers optimal conditions for the development of a highly specialized technical solution to reduce patient falls. Beside the analysis of the requirements for a technical solution, the cooperation helped to adapt the system to a daily clinical routine.

Among others, a 1.5-year study launched in 2013 (cf. Section 1.2), revealed information on clinical structures, potential optimizations and other requirements. Specialists from hospital IT, nursing and diagnostics, as well as human resource management and hygiene, made it possible to derive numerous requirements (cf. Section 2.2) to develop a system that can be used under clinical conditions—the INBED system as described by this article.

The main component of this system is the INBED wearable, which is a functional, small and cost-effective bed-exit sensor system. It fulfils all of the high-specific properties indicated for the use case and is attached directly to the patient. We described the architecture of the INBED wearable, the software implementation as well as the functionality of the entire system, in detail. Extensive testing shows that the system can be utilized to reliably detect several events such as rising, falling or restlessness. Together with the functionality tests, the effectiveness and suitability for daily use of the system has been shown.

In sum, it is possible to design and create a cost-effective and modular fall prevention system for clinical use under the stated conditions. The highly specialized, clinically applicable, INBED system offers the possibility to support professional care in the reduction of patient injuries caused by falls; thus, strengthening the patient’s sense of safety.

### 4.1. Future Work

Further integration of other sensors, e.g., gyroscopes, is planned to enhance the detection of events. Another goal is to further optimize internal processes by adding the possibility to adapt individual configuration values remotely. As the INBED system is operational, a multi-centric, randomized, long-term study within two geriatric wards will start in summer 2019.

### 4.2. Limitations

The system will not be able to detect all the possible fall events. This is due to the medical definition of falls: “A fall is defined as an event which results in a person coming to rest inadvertently on the ground or floor or other lower level” [38]. This also includes smooth falls from an already low position, e.g., low beds (hight approximate 30 cm) to the floor. We plan to include a gyroscope or an air pressure sensor to overcome this issue.
